# Spatially Resolved Association of Structural Biomarkers on Retinal Function in Non-Exudative Age-Related Macular Degeneration Over 4 Years

**DOI:** 10.1167/iovs.65.4.45

**Published:** 2024-04-30

**Authors:** Marlene Saßmannshausen, Senem Döngelci, Marc Vaisband, Leon von der Emde, Kenneth R. Sloan, Jan Hasenauer, Frank G. Holz, Steffen Schmitz-Valckenberg, Thomas Ach

**Affiliations:** 1 Department of Ophthalmology, University Hospital Bonn, Bonn, Germany; 2 Life and Medical Sciences Institute, University of Bonn, Bonn, Germany; 3 Department of Internal Medicine III With Haematology, Medical Oncology, Haemostaseology, Infectiology and Rheumatology, Oncologic Center, Salzburg, Austria; 4 Salzburg Cancer Research Institute–Laboratory for Immunological and Molecular Cancer Research (SCRI-LIMCR), Paracelsus Medical University, Salzburg, Austria; 5 Cancer Cluster Salzburg, Salzburg, Austria; 6 Department of Computer Science, University of Alabama at Birmingham, Birmingham, Alabama, United States; 7 Helmholtz Center Munich–German Research Center for Environmental Health, Institute of Computational Biology, Neuherberg, Germany; 8 John A. Moran Eye Center, University of Utah, Salt Lake City, Utah, United States

**Keywords:** retinal layer thickness, fundus-controlled perimetry, HRF, PED, i-RORA, c-RORA

## Abstract

**Purpose:**

To longitudinally assess the impact of high-risk structural biomarkers for natural disease progression in non-exudative age-related macular degeneration (AMD) on spatially resolved mesopic and scotopic fundus-controlled perimetry testing.

**Methods:**

Multimodal retinal imaging data and fundus-controlled perimetry stimuli points were semiautomatically registered according to landmark correspondences at each annual visit over a period of up to 4 years. The presence of sub-RPE drusen, subretinal drusenoid deposits, pigment epithelium detachments (PEDs), hyper-reflective foci (HRF), vitelliform lesions, refractile deposits, and incomplete RPE and outer retinal atrophy (iRORA) and complete RPE and outer retinal atrophy (cRORA) were graded at each stimulus position and visit. Localized retinal layer thicknesses were extracted. Mixed-effect models were used for structure–function correlation.

**Results:**

Fifty-four eyes of 49 patients with non-exudative AMD (mean age, 70.7 ± 9.1 years) and 27 eyes of 27 healthy controls (mean age, 63.4 ± 8.9 years) were included. During study course, presence of PED had the highest functional impact with a mean estimated loss of −1.30 dB (*P* < 0.001) for mesopic and −1.23 dB (*P* < 0.001) for scotopic testing, followed by HRF with −0.89 dB (mesopic, *P* = 0.001) and −0.87 dB (scotopic, *P* = 0.005). Subretinal drusenoid deposits were associated with a stronger visual impairment (mesopic, −0.38 dB; *P* = 0.128; scotopic, −0.37 dB; *P* = 0.172) compared with sub-RPE drusen (−0.22 dB, *P* = 0.0004; −0.18 dB, *P* = 0.006). With development of c-RORA, scotopic retinal sensitivity further significantly decreased (−2.15 dB; *P* = 0.02). Thickening of the RPE–drusen–complex and thinning of the outer nuclear layer negatively impacted spatially resolved retinal sensitivity.

**Conclusions:**

The presence of PED and HRF had the greatest prognostic impact on progressive point-wise sensitivity losses. Higher predominant rod than cone-mediated localized retinal sensitivity losses with early signs of retinal atrophy development indicate photoreceptor preservation as a potential therapeutic target for future interventional AMD trials.

Detailed insights into structure–function correlations in early and intermediate age-related macular degeneration (AMD) are crucial to identify new structural and functional outcome measures for early disease progression because treatment options are only available for late-stage AMD.^1^^–^^3^

Growing availability of highly reproducible and high-resolution multimodal retinal imaging including optical coherence tomography (OCT) enabled the description of various new phenotypes and biomarkers in non-exudative AMD, going beyond the current definitions in the Beckman classification for AMD disease severity.^4^^,^^5^ Longitudinal studies on intermediate AMD could further highlight the prognostic impact of structural phenotypes as subretinal drusenoid deposits (SDDs), hyper-reflective foci (HRF), large pigment epithelium detachment (PED), and retinal layer thicknesses on disease progression.^5^^–^^8^ Subsequent studies then aimed to address a remaining knowledge gap of structure–function correlation in early and intermediate AMD using dark adaptation time, low-luminance visual deficit, and fundus-controlled perimetry (FCP) testing.^9^^–^^11^ It is now known that the presence of SDD has a greater impact on rod function owing to impaired scotopic retinal sensitivity or dark adaptation time than the presence of sub-RPE drusen^12^^–^^14^ and further correlates with a pronounced visual decline owing to the development of outer retinal degeneration over time.^15^^,^^16^

In a more recent study by Reiter et al.^17^ in eyes with non-exudative AMD, spatially resolved analysis exhibited a direct correlation of both drusen and HRF volume with decreased mesopic FCP sensitivity, with overall higher estimates found for HRF than drusen volume.

However, previous structure–function correlations focused predominantly on the presence of single structural biomarkers, whereas in a real-world clinical setting, early and intermediate AMD eyes present with much more heterogeneity, that is, multiple structural biomarkers simultaneously present within one eye. However, it remains challenging to estimate in which proportions each individual structural parameter contributes to functional decline and to risk for AMD progression over time.

Therefore, longitudinal multimodal high-resolution retinal imaging studies assessing the long-term impact of various structural biomarkers on retinal function are of immense importance in AMD research. In this context, the aim of this study was to examine structural biomarkers in the course of disease progression longitudinally. We correlate the presence and change of these biomarkers as assessed by multimodal retinal imaging data to mesopic and scotopic FCP retinal sensitivity testing over a longitudinal period with annual follow-up visits.

## Methods

### Subjects

This non-interventional natural history study was conducted at the Department of Ophthalmology, University Hospital Bonn, Germany, between December 2015 and September 2022. The study protocol was approved by the Ethics committee at the University Hospital Bonn (#125/14), and all study procedures were conducted in accordance with the Tenets of the Declaration of Helsinki. Informed consent was obtained from all study participants after detailed explanation of the study's purpose, procedures, and potential consequences of participation.

For study eligibility, according to the Beckman classification, patients had to be diagnosed with the presence of large (>125 µm) sub-RPE drusen and/or any AMD pigmentary abnormalities as confirmed by color fundus photography (CFP), near-infrared reflectance (IR), or SD-OCT imaging in either eye.^4^ Patients with predominant presence of SDDs in absence of sub-RPE drusen were not included in this study. Only study subjects with clear optic media, a visual acuity of a logMAR of at least 0.2 as well as stable fixation were enrolled in both patient and control groups. In addition, participants with refractive errors exceeding 3 diopters spherical equivalent, any indications of anterior segment diseases, presence of geographic atrophy identified as a lesion of well-demarcated hypoautofluorescence of 0.1 mm² or greater on fundus autofluorescence (FAF) imaging, presence of complete RPE and outer retinal atrophy (cRORA) at baseline visit based on the definition provided by the Classification of Atrophy Meeting group criteria,^18^ macular neovascularization, diabetic retinopathy, glaucoma, inflammatory retinal diseases, prior laser treatments, or a history of prior intraocular surgery except for cataract surgery more than 3 months earlier were excluded. If both eyes were eligible for study inclusion, both eyes were included. Healthy participants without any signs of current or previous ocular diseases served as controls to support the transformation of retinal layer thicknesses and spatially resolved sensitivity data in terms of deviations from the normal.

### Retinal Imaging Protocol

After pupil dilation (with 0.5% tropicamide and 2.5% phenylephrine), all study participants underwent an extensive and standardized retinal imaging protocol utilizing the high-speed combined and simultaneous confocal scanning laser ophthalmoscopy and SD-OCT system (Spectralis HRA+OCT, Heidelberg Engineering, Heidelberg, Germany; digital imaging resolution 768 × 768 pixels). The standardized imaging procedure encompassed various imaging modalities including IR (30° × 30° field), blue light FAF (excitation wavelength 488 nm, emission wavelength of 500–800 nm, minimum of 15 frames), a single horizontal and vertical combined confocal scanning laser ophthalmoscopy and SD-OCT scan through the fovea (30° field, automated real-time mode, minimum of 9 frames), a raster SD-OCT scan (30° × 25°, automated real-time mode, minimum of 9 frames, 61 B-scans with a distance of 120 µm), as well as CFP imaging. For OCT imaging, the follow-up mode was applied at all visits as provided in the device's imaging platform (Heyex, Heidelberg Engineering).

### FCP

Detailed spatially resolved functional testing was performed using FCP in patients and controls according to previously established protocols ^19^^,^^20^ under mesopic (Goldmann size III, retinal area 0.43°, 200 ms duration, 4-2 strategy, background luminance 1.27 cd/m², 3° radius and 1-pixel fixation ring) and scotopic conditions (Goldmann size V, retinal area 1.7°, 200 ms, 4-2 strategy, background luminance 0.0032 cd/m², 3° radius, and 1-pixel fixation ring) of the macular retina using the Nidek MP-1S (Nidek Technologies, Padova, Italy) with a 56-stimuli testing point grid (10° x 10°) centered on the fovea. Before scotopic testing patients were dark adapted for 30 minutes. Scotopic examination was performed for probing predominantly rod than cone–mediated retinal function, and mesopic served for both rod and cone–mediated retinal function. All participants underwent a test run before the main study examination. Using the MP-1S device, a filter selection test was performed before the scotopic examination to extend the dynamic range of threshold values (neutral density [ND] filter 2.0 log unit, 1.0 log unit, and 0.0 log unit). Owing to a different dynamic range of threshold values, no direct comparison of sensitivity values between ND filters can be performed. For the same reason, values cannot be compared between different FCP devices. In patients, FCP testing was performed at each annual follow-up visit. No ND filter was needed for mesopic testing, accounting for both rod- and cone-mediated retinal function. For the current analysis, only patients and controls with the selection filter ND of 2.0 were included. Patients were excluded from further follow-up visits in case of a progression to any type of macular neovascularization or a ND filter change. Eyes that were once excluded at a follow-up visit were not considered for a follow-up examination at a later time point.

### Retinal Layers Thickness Analysis

For analyzing multiple retinal layer thicknesses, volumetric SD-OCT imaging data were segmented automatically using the device's software tool (Spectralis Viewer Module 6.3.2.0, Heidelberg Engineering Eye Explorer). Semiautomatic segmentation was carefully reviewed in each of the 61 B-scans SD-OCT at each visit and manual corrected if needed.

The retinal layers used in this study were defined following the retinal layer definitions as proposed by Staurenghi et al.^21^: the retinal nerve fiber layer (RNFL) extended from the internal limiting membrane to the boundary between the RNFL and ganglion cell layer (GCL). The GCL spanned from the border of the RNFL and GCL to the border between the GCL and inner plexiform layer (IPL). The IPL encompassed from the GCL/IPL boundary to the border between the IPL and inner nuclear layer. The outer plexiform layer (OPL) covered the space between the inner nuclear layer/OPL border and the OPL/outer nuclear layer (ONL) border. The ONL extended from the OPL/ONL border to the external limiting membrane, including the Henle fiber layer as per the classification by Sadigh et al.^22^ The inner photoreceptor segments were defined as the region between the external limiting membrane and OCT hyper-reflective band 2 (ellipsoid zone), whereas the outer photoreceptor segments extended from the ellipsoid zone to OCT hyper-reflective band 3 (interdigitation zone) or the RPE. The RPE drusen-complex (RPEDC) encompassed from the apex of the RPE layer to Bruch's membrane, including sub-RPE drusen, RPD, basal linear deposits, or basal laminar deposits, if present, as described previously.^23^^,^^24^ Thickness maps for each retinal layer were generated as volumetric en face image maps and exported as tab-delimited files for subsequent analysis using Fiji, ImageJ (U.S. National Institutes of Health, Bethesda, MD, USA).^25^

### Point-Wise Topographic Correlation of Retinal Structure With Retinal Function

**Figure 1.**
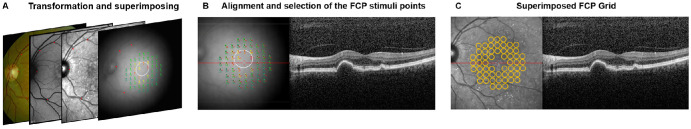
Registration process of retinal imaging data. En face infrared (IR) image of the fundus-controlled perimetry (FCP) were resized and aligned to the en face IR of the SD-OCT image according to vessel-bifurcations, the optic disc position and the foveal position (**A**). After the alignment of retina imaging data, FCP stimuli points were selected and (**B**) superimposed on all prior aligned en face images (**C**). A detailed qualitative and quantitative grading was then performed at each of the 56 FCP stimuli positions in a multimodal retinal imaging approach.

Volumetric en face retinal layer thickness maps were registered to the SLO en face image of the SD-OCT image utilizing a custom-developed Fiji, ImageJ plugin (CreativeComputation, KS, accessible at https://sites.imagej.net/CreativeComputation/). This registration process relied on (1) the identification of vessel bifurcations, (2) the precise determination of the foveal pit characterized by the absence of the inner retinal layers on the central SD-OCT scan, and (3) the optic nerve position. Retinal data from the right eyes were mirrored to left eyes. Next, the position of each FCP stimulus location was aligned to the en face IR of the SD-OCT using another customized ImageJ plugin (Register MP Plugin, https://sites.imagej.net/CreativeComputation/). Figure 1 shows a graphical illustration of the image registration process. Retinal layer thicknesses at the site of each stimulus location and area for scotopic (Goldmann size V; retinal area, 1.7°) test stimuli were then extracted from the corresponding volumetric en face retinal layer thickness map. Further, a qualitative grading on the presence of structural biomarkers was performed at each FCP stimuli location (see the following paragraph).

### Qualitative Grading of Structural Biomarkers at FCP Stimuli Points

A detailed and standardized spatially resolved grading on the presence of structural biomarkers at each FCP stimuli location was performed by two medical readers (M.S., S.D.) in a multimodal retinal imaging approach. All study eyes with iAMD were graded regarding the presence of sub-RPE drusen, SDD, HRF, vitelliform material, PED, refractile deposits and lesions of an incomplete (i-) RORA and cRORA.

In accordance to a previous published study protocol,^26^ sub-RPE drusen were defined as deposits of extracellular material beneath the RPE layer appearing as yellowish–white in CFP and detectable as sub-RPE elevation in SD-OCT imaging.^27^ SDDs were defined as a pattern of oval or roundish irregularities in either IR or FAF imaging corresponding with at least five lesions of hyper-reflective abnormalities or elevations above the RPE–Bruch's membrane in SD-OCT.^12^^,^^28^ HRF are designated as well-circumscribed lesions in proximity to drusen, a thickness of at least one-third of the Bruch's membrane–RPE band being detached from the underlying RPE layer with a reflectivity similar to the RPE layer.^29^^,^^30^ Vitelliform material was defined as an accumulation of hyper-reflective, amorphous material confined to the subretinal space and on top of a sub-RPE druse in SD-OCT imaging, typically associated with an increased FAF image signal.^31^ Lesions of a PED are characterized as an elevation of the RPE layer with a basal diameter of 1000 µm or more and a height of 200 µm or more, as measured from the inner edge of Bruch's membrane in the outer edge of the RPE band in SD-OCT imaging.^32^ Refractile deposits are characterized by either a laminar intense hyperreflectivity (>100 µm) at the level of Bruch's membrane or a pyramidal structure on the outer retina (ghost drusen) in SD-OCT.^33^ Further, a glistening and yellow shining appearance by CFP, a hyper-reflective signal alteration on IR, and/or a mildly increased or mottled FAF signal determined the presence of any refractile lesion.^34^^,^^35^ The presence of iRORA is defined in the OCT as (1) a region of signal hypertransmission into the choroid (<250 µm), (2) a corresponding zone of attenuation or disruption of the RPE (<250 µm), and (3) evidence of overlying photoreceptor degeneration in an overall absence of an RPE tear, whereas in cRORA the aforementioned criteria exceed a dimension of 250 µm.^18^

### Statistical Analyses

Statistical analysis was performed using R (R core Team, version 4.2.1). For FCP analysis, data were normalized in terms of point-wise sensitivity deviation (given in decibels [dB]) from the normative mean of the control group. Therefore, negative values signify a sensitivity loss and positive values supra-normal sensitivity values. For the retinal layer thickness analysis, thicknesses were normalized to *z* scores with respect to the mean and standard deviation of retinal layer thicknesses at the equivalent position in normal age-matched controls.

A *P* value of less than 0.05 was determined to be statistically significant. To investigate associations between structural biomarkers including retinal layer thicknesses to mesopic and scotopic sensitivity testing, we employed a linear mixed-effect model (as implemented in the lmerTest R package),^36^ accounting for a number of different structural biomarkers as exogenous, and scotopic and mesopic sensitivities as endogenous variables. To account for interpatient, intervisit, and grid point correlation, patient ID, patient visit, and grid points were included as random effects.

To capture the longitudinal dynamics in both structural biomarkers and function, we calculated point-wise sensitivity differences between subsequent visits of patients. To investigate structural associations, we then again used a linear mixed-effect model, but this time using the change in function as endogenous variable. The exogenous variables remained the same as in the initial analysis, with the addition of the number of years elapsed between visits.

## Results

### Demographics

Fifty-four eyes of 49 patients with iAMD (mean age, 70.7 ± 9.1 years; median, 73 years, 0.25–0.75; interquartile range, 66.5–78.0 years; 25 females; 14 pseudophakic eyes) and 27 eyes of 27 healthy subjects (63.4 ± 8.9 years; median, 63 years, 0.25–0.75, interquartile range, 56.5–70 years; 18 females; 3 pseudophakic eyes) were included in this study. At baseline visit, the mean best-corrected visual acuity (BCVA) was 0.07 ± 0.1 logMAR (mean Snellen equivalent, 20/25) and low-luminance deficit was 0.34 ± 0.14 logMAR (mean Snellen equivalent, 20/40) in patients. In controls, the mean BCVA was 0.03 ± 0.07 logMAR (mean Snellen equivalent, 20/25) (see Table 1). The median follow-up time was 11.7 months (range, 7–17 months) with a median number of three follow-up visits per patient (range, 0–4 visits).

**Table 1.** Study Cohort Characteristics

	Study Cohort	Control Group
No. of patients	49	27
No. of study eyes	54	27
Mean age (years) at baseline visit (min–max)	70.7 (45–85)	63.4 (50–81)
Follow-up visits mean (range)	3 (0–4)	–
Mean follow-up time (months)	11.7	–
Gender (male)	24	21
Eye (OD)	28	14
Pseudophakic eyes at baseline visit	14	3
BCVA (logMAR) at baseline visit	0.07	0.03
LLVA (logMAR) at baseline visit	0.40	0.29
LLVA deficit (logMAR) at baseline visit	0.33	0.26

BCVA, best-corrected visual acuity; LLVA, low-luminance visual acuity; LLVA deficit, low-luminance visual acuity deficit.

Results for visual acuity are presented as means.

Follow-up examinations were performed at month 12 in 39 eyes, at month 24 in 35, at month 36 in 28, and at month 48 in 24. Detailed results of patients and control demographics, and the detailed number of study eyes available at each follow-up visit as well as detailed reasons for study exits are given in Table 1 and Supplementary Table S1.

### Impact of Structural Biomarkers on Mesopic and Scotopic Retinal Sensitivity Testing

Within non-exudative AMD study eyes, the highest impact of structural biomarkers on retinal sensitivity was detected at retinal locations in presence of PED with a mean estimate of functional decline by −1.30 dB for mesopic and −1.23 dB for scotopic testing, followed by presence of HRF with an overall estimated mesopic loss −0.89 dB *P* < 0.0001 and scotopic loss of −0.87 dB (*P* = 0.0005).

Comparing drusen phenotypes, the presence of SDD was associated with an overall stronger functional decline (−0.38 dB [*P* = 0.128] for mesopic and −0.37 dB [*P* = 0.172] for scotopic testing) than the presence of sub-RPE drusen (−0.22 dB [*P* = 0.0004] for mesopic and −0.18 dB [*P* = 0.006] for scotopic). No significant associations on mesopic and scotopic retinal sensitivity testings were exhibited in presence of vitelliform material (−0.97 dB [*P* = 0.008] for mesopic and −0.66 dB [*P* = 0.095] for scotopic) or refractile deposits (−0.63 dB [*P* = 0.227] and 0.37 dB [*P* = 0.522]).

At FCP stimuli positions with evidence for an early progression toward retinal atrophy development, functional impairment in presence of iRORA lesions was higher for scotopic testing (estimate, −0.86 dB; *P* = 0.004) as compared with mesopic retinal function (estimate, −0.66 dB; *P* = 0.017).

**Figure 2.**
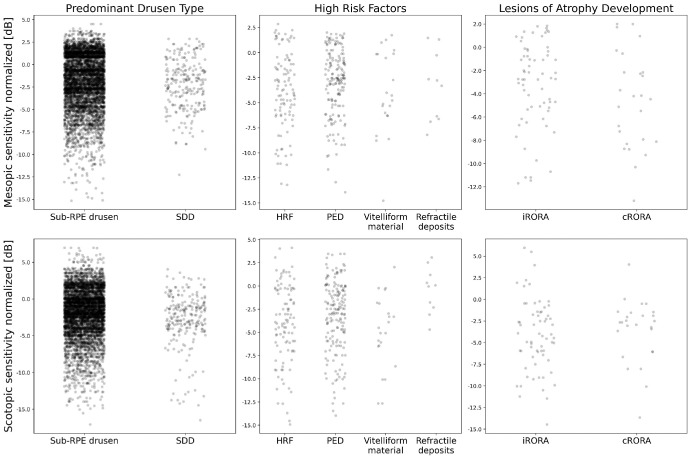
Scatter plots of localized mesopic and scotopic retinal sensitivity in presence of structural high-risk factors. Scatter plots for mesopic (*first* *row*) and scotopic retinal (*second row*) sensitivity testing in dependence of the predominant drusen phenotype (*first column*), other high-risk structural phenotypes including hyperreflective foci (HRF), pigment-epithelium detachment (PED), vitelliform material and refractile deposits (*second column*), as well as for lesions of retinal atrophy development (*third column*) in terms of iRORA and cRORA. All data are given as differences to the normative mean of controls. The horizontal axis of each structural biomarker is given as a jitter for the simplified visibility of individual data points. incomplete (i-) and complete (c-) retinal pigment epithelium and outer retinal atrophy (RORA).

For retinal areas with c-RORA, the estimates were found to be −1.35 dB (*P* = 0.002) for mesopic and 0.47 dB (*P* = 0.327) for scotopic testing. For detailed results, see Table 2 and Figure 2. Detailed information on structural biomarkers at each study visit is given in Supplementary Table S2.

**Table 2.** Impact of Structural Biomarkers on Retinal Sensitivity

	Mesopic				Scotopic			
Parameter	Coefficient Estimates [in dB]	SE	95% CI	*P* Value	Coefficient Estimates (dB)	SE	95% CI	*P* Value
Intercept	**5.06**	**1.45**	**[2.18 to 7.89]**	**0.001**	1.52	1.92	[−2.43 to 5.36]	0.430
Age	−**0.09**	**0.02**	**[**−**0.13 to** −**0.05]**	**<0.0001**	−0.04	0.03	[−0.10 to 0.01]	0.090
Pseudophakic	0.30	0.43	[−1.13 to 0.54]	0.495	0.03	0.56	[−1.06 to 1.11]	0.956
Sub-RPE drusen	−**0.22**	**0.06**	**[**−**0.34 to** −**0.10]**	**0.0004**	−**0.18**	**0.07**	**[**−**0.32 to** −**0.05]**	**0.006**
SDD	−0.38	0.25	[−0.86 to 0.11]	0.128	−0.37	0.27	[−0.90 to 0.16]	0.172
PED	−**1.30**	**0.18**	**[**−**1.65 to** −**0.94]**	**<0.0001**	−**1.23**	**0.20**	**[**−**1.62 to** −**0.85]**	**<0.0001**
Vitelliform material	−**0.97**	**0.36**	**[**−**1.68 to** −**0.26]**	**0.008**	−0.66	0.39	[−1.43 to 0.11]	0.095
HRF	−**0.89**	**0.23**	**[**−**1.34 to** −**0.44]**	**0.0001**	−**0.87**	**0.25**	**[**−**1.36 to** −**0.38]**	**0.0005**
Refractile deposits	−0.63	0.53	[−0.40 to 1.67]	0.227	0.37	0.57	[−0.75 to 1.49]	0.522
iRORA	−**0.66**	**0.28**	**[**−**1.20 to** −**0.12]**	**0.017**	−**0.86**	**0.30**	**[**−**1.45 to** −**0.28]**	**0.004**
cRORA	−**1.35**	**0.44**	**[**−**2.23 to** −**0.48]**	**0.002**	0.47	0.48	[−0.47 to 1.42]	0.327

Note that the SD-OCT thickness data were corrected to *Z* scores and the slopes for fundus-controlled perimetry (FCP) sensitivity results to the normative means of the control group. For example, in mesopic sensitivity testing, eyes with iRORA revealed an overall lower sensitivity threshold by −0.785 dB as compared with nonaffected retina areas. Significant values are presented in bold.

### Inter-Visit Analysis

**Figure 3.**
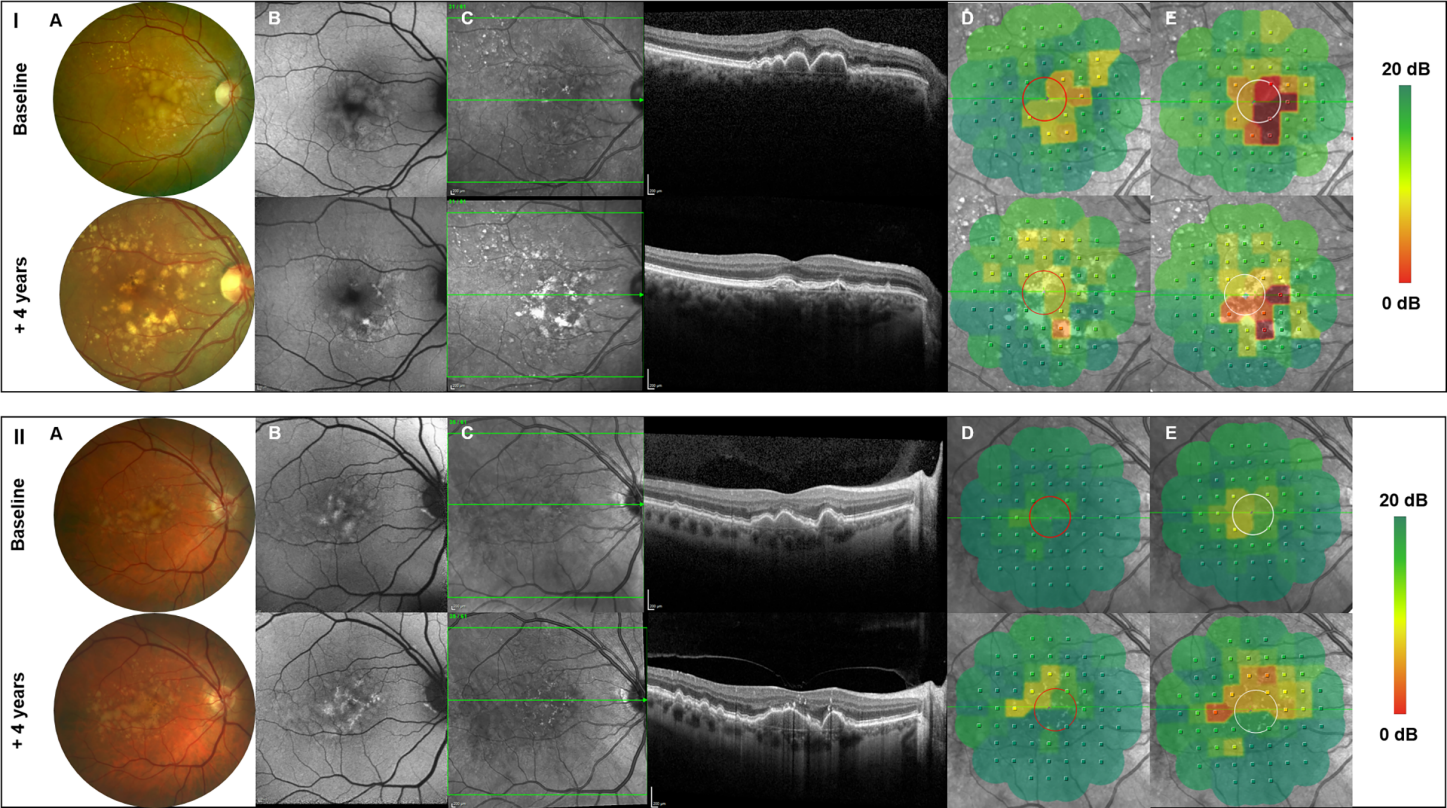
Longitudinal multimodal retinal imaging and mesopic as well as scotopic FCP testing of two representative study patients. Two representative patient examples (I and II) of mulimodal retinal imaging including color fundus photograhy (**A**), autofluoresence retinal imaging (**B**), and SD-OCT (**C**) as well as mesopic (**D**) and scotopic (**E**) FCP results at baseline and at the last performed follow-up visit. Over time, there is a regression of large sub-RPE drusen with development of a progressive degeneration of the outer retinal layers and corresponding to a spatially resolved functional decline of both mesopic and scotopic retinal sensitivity.

The inter-visit analysis evaluated the impact of structural biomarkers on spatially resolved retinal sensitivity changes between two visits. Within two visits, the presence of refractile deposits was significantly associated with highest progressive scotopic functional decline by −3.50 dB (*P* = 0.0004), whereas no significant changes were found for mesopic retinal sensitivity testing (−0.13 dB; *P* = 0.882). At retinal locations with the presence of HRF, there was a progressive although not significant decrease of both mesopic (estimate, −0.07 dB; *P* = 0.860) and scotopic (estimate, 0.65 dB; *P* = 0.159) retinal function between visits. With the development of cRORA lesions, the intervisit analysis further exhibited a progressive functional decline by −0.24 dB [*P* = 0.771] for mesopic testing and by −2.15 dB [*P* = 0.020] for scotopic testing between the two study visits. Detailed parameter estimates of structural phenotypes can be found in Table 3. Two representative patient examples with structural alterations and correlating functional impairment over time are given in Figure 3.

**Table 3.** Intervisit Association of Structural Parameters on Retinal Sensitivity

	Mesopic				Scotopic			
Parameter	Coefficient Estimates Change [in dB]	SE	95% CI	*P* Value	Coefficient Estimates Change [in dB]	SE	95% CI	*P* Value
(Intercept)	**2.65**	**1.03**	**[0.65 to 4.56]**	**0.013**	**10.71**	**1.64**	**[4.21 to 16.73]**	**<0.0001**
Age	−**0.04**	**0.01**	**[**−**0.06 to** −**0.01]**	**0.017**	−**0.15**	**0.02**	**[**−**0.24 to** −**0.06]**	**<0.0001**
Time between visits	−**0.49**	**0.21**	**[**−**0.90 to** −**0.08]**	**0.019**	1.64	0.23	[−0.34 to 0.60]	0.481
Pseudophakic	**0.72**	**0.24**	**[0.23 to 1.56]**	**0.003**	0.21	0.30	[−0.37 to 0.77]	0.474
Sub-RPE drusen	0.12	0.09	[−0.06 to 0.29]	0.185	−**0.30**	**0.10**	**[**−**0.50 to** −**0.11]**	**0.003**
SDD	0.03	0.34	[−0.63 to 0.71]	0.929	−0.06	0.38	[−0.81 to 0.68]	0.869
PED	**0.74**	**0.27**	**[0.22 to 1.26]**	**0.005**	0.40	0.29	[−0.18 to 0.96]	0.175
Vitelliform material	0.52	0.63	[−0.70 to 1.75]	0.403	1.17	0.69	[−0.22 to 2.49]	0.088
HRF	−0.07	0.42	[−0.90 to 0.75]	0.860	0.65	0.46	[−0.28 to 1.54]	0.159
Refractile deposits	−0.13	0.90	[−1.89 to 1.63]	0.882	−**3.50**	**0.99**	[−5.44 to −1.57]	**0.0004**
i-RORA	**0.94**	**0.45**	**[0.05 to 1.83]**	**0.040**	0.95	0.50	[0.03 to 1.93]	0.573
c-RORA	−0.24	0.84	[−1.89 to 1.41]	0.771	−**2.15**	**0.92**	**[**−**3.96 to** −**0.34]**	**0.020**
RNFL [*z* scores]	−**0.07**	**0.04**	**[**−**0.153 to** −**0.004]**	**0.038**	−0.01	0.04	[−0.09 to 0.07]	0.845
GCL [*z* scores]	−0.03	0.04	[−0.103 to 0.042]	0.410	0.04	0.04	[−0.04 to 0.12]	0.369
IPL [*z* scores]	−0.01	0.05	[−0.103 to 0.090]	0.897	−0.02	0.05	[−0.12 to 0.09]	0.770
INL [*z* scores]	0.06	0.04	[−0.021 to 0.139]	0.144	0.02	0.04	[−0.06 to 0.11]	0.596
OPL [*z* scores]	0.02	0.04	[−0.039 to 0.077]	0.518	0.02	0.03	[−0.04 to 0.09]	0.539
ONL [*z* scores]	0.07	0.04	[0.016 to 0.151]	0.115	0.003	0.05	[−0.09 to 0.10]	0.947
IS [*z* scores]	−0.001	0.001	[−0.003 to 0.001]	0.175	−0.0001	0.001	[−0.001 to 0.002]	0.861
OS [*z* scores]	0.002	0.03	[−0.054 to 0.059]	0.940	−0.01	0.03	[−0.07 to 0.05]	0.696
RPEDC [*z* scores]	−**0.05**	**0.006**	**[**−**0.058 to** −**0.034]**	**<0.0001**	−0.0002	0.01	[−0.01 to 0.02]	0.967

GCL, ganglion cell layer; HRF, hyperreflective foci; INL, inner nuclear layer; IPL, inner plexiform layer; IS/OS, inner and outer photoreceptor segments; ONL, outer nuclear layer; OPL, outer plexiform layer; PED, pigment epithelium detachment; RNFL, retinal nerve fibre layer; RORA, incomplete (i-) and complete (c-) outer retinal atrophy; RPEDC, retinal pigment epithelium drusen complex; SDD, subretinal drusenoid deposits.

Note that the SD-OCT thickness data were corrected to *z* scores and the slopes for FCP sensitivity results to the normative means of the control group. For example, in mesopic sensitivity testing, eyes with SDD reveal an overall lower sensitivity threshold by −0.10 dB compared with retinal areas without drusen presence. Significant values are presented in bold.

With the inclusion of the retinal layer thickness as additional covariates, we found retinal layer thicknesses of the RPEDC and the outer retinal layers, including the ONL and inner photoreceptor segments as most relevant for a preserved spatially resolved retinal sensitivity.

For mesopic and scotopic testing, respectively, the estimated model coefficients for the RPEDC, for example, were found to be −0.046 and −0.0002. This result means that a local thickening of the RPEDC layer by 1 SD, corresponding with an average change of +1.98 µm, would be associated with an estimated sensitivity loss of −0.046 dB for mesopic testing (*P* < 0.0001) and of −0.0002 dB for scotopic testing (*P* = 0.967) within 1 year. Detailed estimates for retinal layer thickness as well as estimated retinal layer thickness changes per year are given in Table 3 and Supplementary Table S3.

## Discussion

This detailed study is the first to consider the impact of multiple established structural biomarkers on spatially resolved mesopic and scotopic retinal sensitivity. Our study reports multimodal and longitudinally data from eyes with non-exudative AMD, with annual follow-ups over a period of up to 4 years. With the aid of a custom ImageJ analysis workflow, we were able to correlate structural alterations precisely (as seen in multimodal retinal imaging) to the topographic position and stimuli size of each FCP testing point at each study visit.

Within non-exudative AMD study eyes, the overall greatest impact on mesopic and scotopic retinal sensitivity was detected at retinal locations with the presence of PED and HRF, whereas the least impairment on retinal function was exhibited in presence of refractile deposits for both sensitivity testings, despite high standard errors. Although detailed data on spatially resolved structure–function correlations considering concomitant structural phenotypes in non-exudative AMD eyes are limited, our results underline previous findings by Kitano et al.,^37^ revealing a significantly deteriorated mesopic retinal sensitivity at retinal locations with the presence of drusenoid or serous PED lesions without evidence of macular neovascularization. Furthermore, our results support findings by Echols et al.^38^ reporting a significant correlation between HRF presence and increasing HRF count to a progressive impairment of retinal function, including greater losses of scotopic than mesopic function as assessed by FCP testing. In non-exudative AMD study eyes, Reiter et al.^17^ just recently showed a decreased mesopic retinal sensitivity of −1.422 dB when HRF are present, which was—despite the use of different FCP devices and testing protocols—overall higher compared with the mesopic functional impairment of −0.893 dB (*P* = 0.0001) in HRF presence in our study. Interestingly, in both the work by Reiter et al.^17^ and our study, localized mesopic retinal sensitivity was stronger affected by the presence of HRF than sub-RPE drusen, although the overall impact of sub-RPE drusen presence on retinal sensitivity remained significant. These concurring results support the important role of HRF as a high-risk factors for disease progression and functional decline in non-exudative AMD, as reported previously.^6^^,^^39^^,^^40^

Comparing overall sensitivity losses in the presence of different drusen phenotypes, our results are in line with preceding studies observing a stronger functional impairment of patients with presence of SDDs compared with sub-RPE drusen.^19^^,^^20^ We found no significant differences in functional estimates for mesopic and scotopic retinal function in the presence of SDDs. This finding can most likely be attributed to the exclusion of study eyes with predominant SDDs presence at baseline. Nevertheless, a number of previous studies showed the predominant topographical and functional correlation of SDDs with rods rather than cones.^19^^,^^41^^–^^43^ Other studies also highlighted the important role of xanthophyll carotenoid pigments for cone protection at foveal and perifoveal regions and that the predominantly foveally located Müller glia is sought to be a major xanthophyll reservoir.^44^^,^^45^

Regarding refractile deposits or vitelliform material, no significant associations with retinal function were found, most likely owing the overall low prevalence of these deposits in our study eyes. Nevertheless, previous studies have reported vitelliform material and refractile deposits as important precursor lesions for atrophy development.^5^^,^^29^^,^^35^

For structural biomarkers of early atrophy development, the greatest sensitivity losses were found for mesopic testing at retinal locations with presence of c-RORA, whereas scotopic retinal sensitivity loss was only significant with the presence of i-RORA lesions.

These results are also in line with previous clinical as well as histological–clinical correlation studies, which suggested that rod dysfunction precedes cone dysfunction in AMD disease, probably owing to a shortening and ongoing degeneration of rod outer segments with disease progression, while cones remain resilient.^46^^,^^47^ Whether dysfunctional RPE and/or choriocapillaris flow deficits are the primary factor leading to a progressive photoreceptor impairment exceeding normal aging processes is still under debate.^48^

Regarding the intervisit analysis, there was a significant progressive functional decline for scotopic testing at c-RORA lesions only, whereas for i-RORA lesions, sensitivity results varied at a low significance level for mesopic testing between follow-up visits. These results further suggest that there is a high degree of variability of functional data in patients over time. For a degenerative retinal disease as AMD, where the manifestation of first structural alterations can often take years or decades, even a follow-up period of up to 4 years with the available FCP device is too short to observe significant changes over time. Moreover, the classification into intermediate AMD according to Ferris et al.^4^ may include several different manifestation stages of disease toward atrophy development. Thus, study eyes with only slight structural alterations at baseline visit seem to have no significant changes over time compared with those developing iRORA or cRORA lesions.

Although FCP testing allows to discriminate between functionally impaired and nonimpaired retinal locations in non-exudative AMD cases, the currently applied FCP technology does not seem to be capable enough to fully differentiate slight sensitivity changes between all underlying structural biomarkers over time. At the same time, localized structural alterations might exceed the applied FCP stimuli size or the FCP stimuli is not fully centered on the tested retinal lesion; thus, it may remain challenging to test spatially resolved visual impairment owing to the isolated presence of a single structural biomarker.

Therefore, future longitudinal studies with larger study cohorts including detailed structural and functional testings are needed to also correlate these findings to patient-reported outcome measures, as performed in the longitudinal study cohort of the European multicenter MACUSTAR study on intermediate AMD.^49^ And, with the implementation of patient-tailored FCP testing grids positioned at high-risk retinal locations and machine learning approaches on large multicenter datasets, even more information on the risk-stratified impact of structural biomarkers on retinal function can be gathered.^50^^,^^51^

Our study has several limitations. First, the sample size of included patients with non-exudative AMD is small, especially at follow-up visits owing to several reasons for study exclusion over time. This factor led to potentially low statistical power in detecting differences of retinal sensitivity over time and needs to be carefully considered in data interpretation. In contrast, this is—to the authors’ best knowledge—the first study performing a detailed structure–function analysis of several simultaneously present structural biomarkers in a longitudinal study setting with standardized high-quality imaging and functional testing protocols, as well as a spatially resolved grading of various established structural AMD biomarkers. Certainly, further longitudinal observational studies with larger studies cohorts and highly standardized grading protocols, as well as refine structure–function correlations in early AMD studies eyes (e.g., ALSTAR2 [Alabama Study on Early Age-related Macular Degeneration 2], MACUSTAR, and PINNACLE study) are needed to confirm our findings.

Second, despite of the inclusion of study eyes according to the current Beckman classification for intermediate AMD, there remains a certain heterogeneity of study eyes, because single-study eyes already had evidence of iRORA at baseline visit. Third, there are several limitations regarding the use of the microperimetry device; the previously reported ceiling effect, particularly for mesopic testing, owing to a limited threshold range^52^ and the inability to compare sensitivity values between visits in case of a ND filter change led to an exclusion of patients from further longitudinal analysis. Fourth, precise correlation of multimodal retinal imaging data to the FCP stimuli grid over time is crucial for reliable results. Slight shifts of the FCP grids over time cannot be ruled out completely, decreasing the comparability of measurements. To mitigate this limitation, the ImageJ plugin that we developed served as the basis for our analysis platform and enabled the alignment of multimodal retinal images and the SLO image of the FCP device. Fifth, no OCT angiography imaging was performed at the beginning of the study to check for and exclude quiescent choroidal neovascularization underneath a PED lesion. Finally, only a few study eyes have developed exudation over time. The onset of functional impairment in these patients could differ from eyes progressing toward non-exudative late-stage AMD.

The strengths of this study include the prospective longitudinal study design over as many as 4 years in patients with non-exudative AMD with a highly standardized imaging and functional testing protocol performed at each annual follow-up visit and the ImageJ platform based workflow to align structural to functional datasets over time, as well as the detailed and standardized grading of spatially resolved structural biomarkers according to established reading center standards.

In summary, within this study, the presence of PED lesions and HRF were found to have the strongest associations with mesopic and scotopic functional decline in non-exudative AMD. Over time, mesopic dysfunction was following previous predominant rod-mediated function loss, whereas the overall highest functional loss was detected with development of cRORA lesions. Future multicenter longitudinal natural history studies are needed to obtain spatially resolved functional findings in a larger non-exudative AMD study cohort, as well as to investigate to what extent longitudinal functional decline can be estimated from retinal structure, for example, the presence and change of structural biomarkers alone.
